# Early-Life Factors as Predictors of Age-Associated Deficit Accumulation Across 17 Years From Midlife Into Old Age

**DOI:** 10.1093/gerona/glac007

**Published:** 2022-01-09

**Authors:** Markus J Haapanen, Juulia Jylhävä, Lauri Kortelainen, Tuija M Mikkola, Minna Salonen, Niko S Wasenius, Eero Kajantie, Johan G Eriksson, Mikaela B von Bonsdorff

**Affiliations:** Folkhälsan Research Center, Helsinki, Finland; Department of General Practice and Primary Health Care, University of Helsinki, Helsinki, Finland; Department of Medical Epidemiology and Biostatistics, Karolinska Institutet, Stockholm, Sweden; Department of Medical Epidemiology and Biostatistics, Karolinska Institutet, Stockholm, Sweden; Faculty of Social Sciences (Health Sciences) and Gerontology Research Center, Tampere University, Tampere, Finland; Folkhälsan Research Center, Helsinki, Finland; Folkhälsan Research Center, Helsinki, Finland; Clinicum, Faculty of Medicine, University of Helsinki, Helsinki, Finland; Department of Public Health and Welfare, Population Health Unit, Finnish Institute for Health and Welfare, Helsinki, Finland; Folkhälsan Research Center, Helsinki, Finland; Department of General Practice and Primary Health Care, University of Helsinki, Helsinki, Finland; Department of Public Health and Welfare, Population Health Unit, Finnish Institute for Health and Welfare, Helsinki, Finland; PEDEGO Research Unit, Medical Research Center Oulu, Oulu University Hospital, University of Oulu, Oulu, Finland; Children’s Hospital, Helsinki University Hospital, University of Helsinki, Helsinki, Finland; Department of Clinical and Molecular Medicine, Norwegian University of Science and Technology, Trondheim, Norway; Folkhälsan Research Center, Helsinki, Finland; Department of General Practice and Primary Health Care, University of Helsinki, Helsinki, Finland; Yong Loo Lin School of Medicine, Department of Obstetrics and Gynecology and Human Potential Translational Research Programme, National University Singapore, Singapore; Singapore Institute for Clinical Sciences (SICS), Agency for Science, Technology and Research (A*STAR), Singapore; Folkhälsan Research Center, Helsinki, Finland; Gerontology Research Center and Faculty of Sport and Health Sciences, University of Jyväskylä, Jyväskylä, Finland

**Keywords:** Birth factors, Frailty, Life course, Risk factors

## Abstract

**Background:**

Early-life exposures have been associated with the risk of frailty in old age. We investigated whether early-life exposures predict the level and rate of change in a frailty index (FI) from midlife into old age.

**Methods:**

A linear mixed model analysis was performed using data from 3 measurement occasions over 17 years in participants from the Helsinki Birth Cohort Study (*n* = 2 000) aged 57–84 years. A 41-item FI was calculated on each occasion. Information on birth size, maternal body mass index (BMI), growth in infancy and childhood, childhood socioeconomic status (SES), and early-life stress (wartime separation from both parents) was obtained from registers and health care records.

**Results:**

At age 57 years the mean FI level was 0.186 and the FI levels increased by 0.34%/year from midlife into old age. Larger body size at birth associated with a slower increase in FI levels from midlife into old age. Per 1 kg greater birth weight the increase in FI levels per year was −0.087 percentage points slower (95% confidence interval = −0.163, −0.011; *p* = 0.026). Higher maternal BMI was associated with a higher offspring FI level in midlife and a slower increase in FI levels into old age. Larger size, faster growth from infancy to childhood, and low SES in childhood were all associated with a lower FI level in midlife but not with its rate of change.

**Conclusions:**

Early-life factors seem to contribute to disparities in frailty from midlife into old age. Early-life factors may identify groups that could benefit from frailty prevention, optimally initiated early in life.

Frailty is a geriatric syndrome that predisposes individuals to adverse health outcomes and hinders recovery from stressors (1). Although it is mostly observed among older adults (2), the origins of frailty can lie in early life, with further contribution from factors throughout the life course. The application of a life course approach (3) to frailty research has increased our understanding of plausible risks associated with physical and socioeconomic factors that occur during gestation, childhood, and adolescence (4–8). Aspects of early development have been shown to associate with multiple later health outcomes through mechanisms including programming, where developing organ systems may be altered during sensitive periods (9). In the Helsinki Birth Cohort Study (HBCS), we have previously shown that those who were smaller in body size at birth conferred greater risk of physical frailty in the seventh decade (6). Fried et al. (10) introduced this categorical definition of frailty where the simultaneous presence of at least 3 of 5 known criteria is required for an individual to be classified as frail. Using this definition in the cohort, boys who experienced accelerated body mass index (BMI) gain in childhood were at increased risk of frailty in old age (4). The risk of frailty was also higher among boys who had been separated from their parents during World War II, that is, who experienced extreme early-life stress (5). In other studies, the risk of frailty was lower among those with higher educational attainment, better neighborhood quality, and better overall health in childhood (7).

Rather than physical frailty, a continuous frailty index (FI) captures frailty as the proportion of acquired health-related deficits (11). Defining frailty in this way, lower socioeconomic position in childhood has been associated with an increased risk of frailty among 50-year-olds (8). Given that frailty is a dynamic process (12), we are aware of no studies to have investigated how earlier life may relate to the trajectory of frailty from younger and healthier age groups into old age. To study this, we tracked the development of an FI measured 3 times among participants aged 57–84 years in the HBCS who also had information on early-life factors comprising gestation, birth, infancy, and childhood. We hypothesize higher levels and faster increases in frailty among participants with a disadvantageous early life—for example, who had been born small, experienced early-life stress, or abnormal growth.

## Materials and Methods

### Study Design

**Figure 1.**
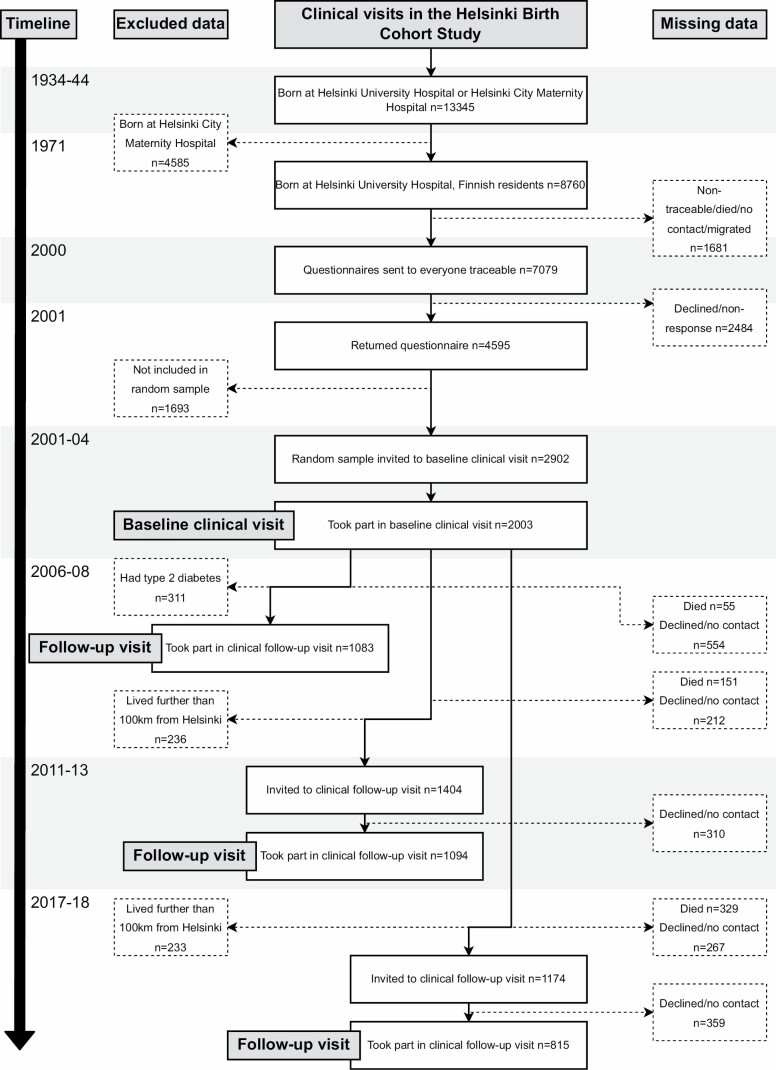
Flowchart of participants in the present study.

The study participants belong to the HBCS and were born at Helsinki University Central Hospital between 1934 and 1944 (13). Figure 1 presents a flowchart of the study population. Unique national personal identification numbers were used to trace participants and link register information about, for example, their health, socioeconomic factors, drug reimbursement, and deaths. Thus far, the participants have attended a maximum of 4 clinical visits between 2001–2018. The present study uses information from the visits conducted between 2001–2004 (*n* = 2 003; mean age = 61.5 years, standard deviation [*SD*] = 2.9 years), 2011–2013 (*n* = 1 094; mean age = 71.1 years; *SD* = 2.7 years), and 2017–2018 (*n* = 815; mean age = 75.9 years; *SD* = 2.7 years). The study was approved by the Ethics Committee of Epidemiology and Public Health of the Hospital District of Helsinki and Uusimaa and that of the National Public Health Institute, Helsinki.

### Deficit Accumulation-Based FI Assessed 3 Times Between 2001–2018

The FI in HBCS is based on the Rockwood deficit accumulation model (11). It was created according to the standard procedure (14) and calculated for each measurement occasion. It included relevant deficits that associate with health status and cover a wide range of systems. We excluded candidate deficits that were uncommon (prevalence <1%), saturated early, or had more than 10% missing data from any single deficit from any of the 3 measurement occasions. We obtained an individual’s FI level by counting the number of deficits and dividing this count by the total number of deficits considered; Supplementary Table 1 presents the 41 included items and their scoring into deficits. We considered symptoms, diseases, disabilities, clinical measurements, and laboratory test results and only included individuals with information on at least 33 of 41 deficits included (ie, deficit count >80% available (14); 99.6% or *n* = 1 995 in 2001–2004; 99.9% or *n* = 1 081 in 2011–2013; 99.1% or *n* = 806 in 2017–2018). A total of 2 000 participants had information on the FI from at least 1 of 3 measurement occasions and constitute the analytical sample of the study. The FI × 100 level of ≥25 was used to indicate the “frail” state (15,16). We assessed the characteristics of the 41-item HBCS–FI by examining its distribution according to age and sex and found it comparable to previous studies (14,17,18).

### Early-Life Factors Comprising Maternal Factors, Body Size at Birth, Childhood Growth, and Socioeconomic Factors

Characteristics of the participants’ mothers included information on body weight and height measured on admission in labor. Gestational age was estimated from the date of the last menstrual period. The participants’ weight and length at birth were retrieved from hospital birth records, after which they were serially measured at child welfare and school health care clinics from infancy to childhood. These data were later obtained from records at the Helsinki City archives. Growth in infancy and childhood was assessed as described previously (4,13). Based on the father’s highest occupational status, childhood socioeconomic status (SES) was coded as manual workers, lower middle class, and upper middle class. The participants’ highest attained occupational status at 5-year intervals was obtained from Statistics Finland between 1970 and 1995 and coded as manual workers, self-employed, lower, and upper officials (19). Information on separations during World War II (20), in which participants were sent abroad unaccompanied by their parents, was obtained from the Finnish National Archives.

### Statistical Methods

Separate linear mixed models were used to examine the associations of early-life factors with FI levels at age 57 years and the rate of change in FI levels from midlife into old age. Age was used as the central time variable and was centered at 57 years (the lowest in the data). All other continuous variables were centered at their mean values. All models were adjusted by adding sex, childhood and adult SES, and their interactions with age to the models. Given potential interrelationships between early-life factors, for example, between size at birth and gestational age, and between maternal BMI and birth weight, models including body size at birth were also adjusted for gestational age and maternal BMI models additionally for birth weight. Interactions between exposure variables and sex on FI were also tested given previously observed sex-depended associations of early-life factors (4,5,21–23). We observed a significant sex interaction only in the model of temporary wartime separation as exposure variable, where also the 3-way interaction of sex * age * separation status was added to the model. Possible U-shaped associations between the variables and FI levels were tested by adding a quadratic term and its interaction with age to the models. If the quadratic term was statistically significant, it was left to the model.

To account for missing data during the study, as a sensitivity analysis, we used a joint modeling approach that takes into account the possibility of missing not at random (MNAR) missing data mechanism in FI values (24). In our joint model, we used the parameterization where the risk of death and frailty level estimated from the linear mixed model were associated. We assumed a parametric, relative risk survival model in which the log baseline hazard function was approximated with B-splines. We used 5 internal knots at equidistant percentiles of the observed event times. The time-constant predictors added to the survival submodel were smoking status, sex, and educational level. The longitudinal models were defined similarly as when the missing at random data mechanism was assumed.

We multiplied the FI by 100 to improve interpretability of the model estimates and treat them as a percentage. For the rate of change, estimates correspond to percentage point (PP) differences of change in FI levels per year. Negative point estimates refer to a lower level of frailty in midlife or slower increase in the rate of change in FI levels from midlife into old age. 95% confidence intervals (CIs) for predictions were calculated using parametric bootstrap. For statistical significance, *p* value of .05 was used. All the analyses were produced in R (25) using packages Ime (26) and ImerTest (27) and the joint models fitted using package JM (28).

## Results

### The FI Level at the Age of 57 Years and the Rate of Change in FI Levels From Midlife Into Old Age

**Figure 2.**
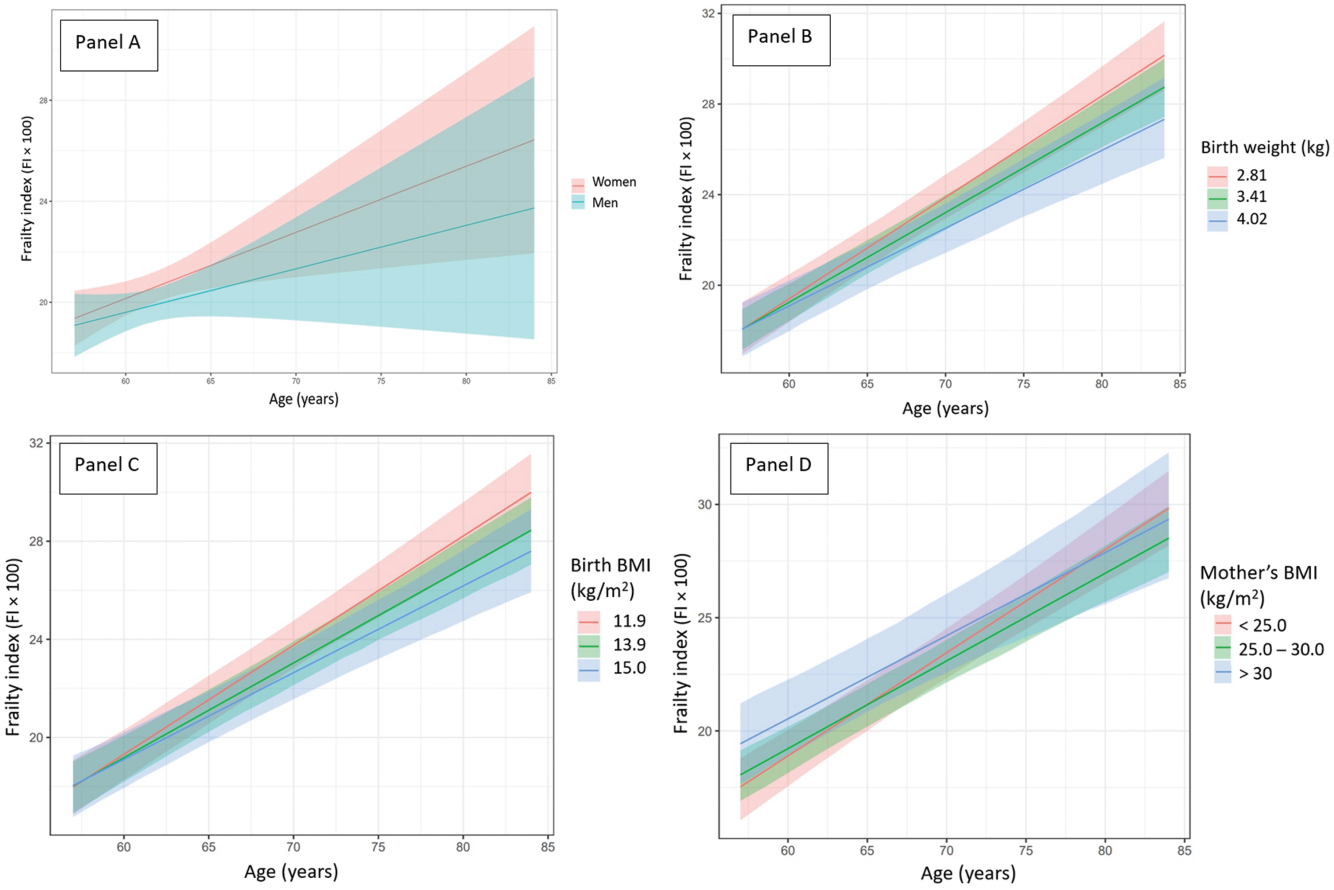
(Panels A–D) Mean frailty index levels (FI × 100) and as a function of age in the Helsinki Birth Cohort Study: shown (A) separately for men and women, (B) according to quantiles 0.1, 0.5, and 0.9 of birth weight which correspond to values 2.81, 3.41, and 4.02 kg of birth weight, (C) according to quantiles 0.1, 0.5, and 0.9 of body mass index (BMI) at birth which correspond to values 11.9, 13.9, and 15.0 kg/m^2^ of BMI at birth, (D) according to groups of maternal BMI (<25.0, ≥25.0 and ≤30.0, >30.0 kg/m^2^).

At the age of 57 years, the mean FI level (FI × 100) was 18.59 (95% CI = 18.08, 19.11; *p* < .001) and it increased by 0.34%/year (95% CI = 0.31, 0.37; *p* < .001) from midlife into old age. Figure 2 panel A shows the mean trajectories of FI levels for men and women from midlife into old age. The FI levels of women were higher than those of men and the rate of change in FI levels was steeper among women than men from midlife into old age.

### Body Size at Birth and the Level and Rate of Change in FI Levels From Midlife Into Old Age

Table 1 and Supplementary Table 2 show the early-life characteristics of the cohort for men and women participating in the baseline clinical visit. Supplementary Table 3 shows these characteristics for invited, lost, and dead participants, as per their status at the clinical follow-up visit. The point estimates of early-life factors predicting the level and rate of change of FI levels from midlife into old age are shown in Table 2. Greater birth weight and BMI were both associated with a less steep rate of change in the FI levels from midlife into old age. One kilogram greater birth weight was associated with −0.087 PP slower (95% CI = −0.163, −0.011; *p* = .026) increase in FI per year. Similarly, 1 kg/m^2^ higher birth BMI was associated with −0.029 PP slower (95% CI = −0.057, −0.001; *p* = .041) increase in FI per year. Figure 2 panels B and C present the FI levels and their rates of change from midlife into old age according to groups of birth weight and BMI and show that the FI levels increased steeper for those who were smaller at birth, that is, who had a lower birth weight or BMI. Reading from Figure 2 panels B and C, the mean FI levels of the biggest-born versus smallest-born groups of birth weight and birth BMI reached the FI level demarcating the “frail” state (FI = 0.25 or FI × 100 = 25 in the figure) on average 5 years earlier (80 vs 75 years). Gestational age, while not associating with the FI level in midlife, showed that per each week longer duration of gestation the respective increase in FI levels was −0.020 PP slower (95% CI = −0.041, −0.001; *p* = .049) per year from midlife into old age. Estimates of Table 2 predictors assuming MNAR sample attrition showed parallel results apart from gestational age and birth BMI, which were attenuated (Supplementary Table 4).

**Table 1.** Characteristics of Participants Participating in Baseline Clinical Measurements

	Total Study Population (*n* = 2 003)	Women (*n* = 1 075)	Men (*n* = 928)
	Mean (*SD*)	Mean (*SD*)	Mean (*SD*)
Birth factors			
Weight (kg)	3.41 (0.49)	3.35 (0.47)	3.48 (0.50)
Length (cm)	50.2 (2.0)	50.0 (1.8)	50.7 (2.1)
BMI (kg/m^2^)	13.41 (1.24)	13.36 (1.22)	13.48 (1.25)
Gestational age (weeks)	40.0 (1.6)	40.1 (1.6)	40.0 (1.5)
Maternal BMI (kg/m^2^)	26.5 (2.9)	26.5 (2.9)	26.5 (3.0)
Wartime separation from both parents during World War II			
Separated, N (%)	268 (14.8)	133 (12.4)	136 (14.7)
Childhood SES			
Manual worker, *N* (%)	1 185 (59.8)	259 (60.8)	175 (52.2)
Lower middle class, *N* (%)	453 (22.9)	95 (22.3)	79 (23.6)
Upper middle class, *N* (%)	343 (17.3)	72 (16.9)	81 (24.2)
Adult SES			
Manual worker, *N* (%)	669 (33.6)	70 (16.3)	114 (33.9)
Self-employed, *N* (%)	186 (9.3)	37 (8.6)	33 (9.8)
Lower official, *N* (%)	853 (42.8)	265 (61.8)	99 (29.5)
Upper official, *N* (%)	286 (14.3)	57 (13.3)	90 (26.8)
Participant characteristics assessed in early old age			
Age (years)	61.5 (2.9)	61.5 (2.8)	61.5 (3.0)
Frailty index			
Baseline measurement occasion in 2001–2004*	0.20 (0.10)	0.21 (0.10)	0.20 (0.10)
Follow-up visit in 2011–2013†	0.21 (0.10)	0.23 (0.10)	0.19 (0.10)
Follow-up visit in 2017–2018‡	0.23 (0.11)	0.24 (0.11)	0.21 (0.10)

*Notes*: BMI = body mass index; *SD* = standard deviation; SES = socioeconomic status.

*n = 1 995.

^†^ *n* = 1 081.

^‡^ *n* = 806.

**Table 2.** One-Unit Increases in Early-Life Factors Predicting Point Estimates of the FI Level at Age 57 Years and the Rate of Change in FI Levels From Midlife Into Old Age

	Level*	95% CI	*p* Value	Rate of Change†	95% CI	*p* Value
Early-life factor¶						
Birth factors						
Weight (kg)‡	−0.001	−1.208, 1.207	.999	−0.087	−0.163, −0.011	.026
Length (cm)‡	−0.077	−0.381, 0.227	.619	−0.016	−0.034, 0.003	.098
BMI (kg/m^2^)‡	0.021	−0.424, 0.466	.926	−0.029	−0.057, −0.001	.041
Gestational age (weeks)	0.118	−0.221, 0.457	.496	−0.020	−0.041, −0.001	.049
Maternal BMI (kg/m^2^)‡	0.263	0.066, 0.460	.009	−0.009	−0.021, 0.003	.134
Maternal BMI (kg/m^2^)‡^,^§	0.218	0.002, 0.435	.048	−0.014	−0.028, −0.002	.026
Wartime separation from both parents during World War II‖						
Separated	2.190	−0.178, 4.558	.071	−0.085	−0.237, 0.067	.275
Separated × female sex	−3.429	−6.670, −0.189	.039	0.211	0.009, 0.414	.041
Childhood SES‖						
Manual worker	2.312	0.889, 3.736	.002	−0.066	−0.150, 0.017	.119
Lower middle class	1.183	−0.438, 2.805	.153	−0.018	−0.114, 0.078	.719
Upper middle class	Ref.			Ref.		

*Notes*: CI = confidence interval; FI = frailty index; BMI = body mass index; SES = socioeconomic status.

*In FI × 100 units, which correspond percentage increases/decreases in FI levels at age 57 years (mean FI level at age 57 years was 0.186).

^†^In percentage points per year from midlife into old age (mean annual rate of change in FI levels from midlife into old age was 0.34%/year).

^‡^Model adjusted with sex, gestational age, childhood and adult SES.

^§^Quadratic term added, model adjusted additionally with birth weight.

^‖^Model adjusted with sex and adult SES.

^¶^Examined individually in separate models.

### Maternal BMI and the Level and Rate of Change in FI Levels From Midlife Into Old Age

Higher maternal BMI was associated with a higher offspring FI level in midlife but with a slower increase in FI levels from midlife into old age when incorporating a quadratic term (Table 2). Per 1 kg/m^2^ higher mother’s BMI the offspring FI level in midlife was 0.263% higher (95% CI = 0.066, 0.460; *p* = .009). When incorporating a quadratic term, we observed a curvilinear association between maternal BMI and a slower increase in FI levels per year from midlife into old age (*p* = .026). Figure 2 panel D presents the mean FI levels and their rates of change in groups of maternal BMI. Reading from the figure, the mean FI levels in midlife were highest among offspring of mothers in the group with the highest BMI (BMI > 30 kg/m^2^), whereas the FI levels increased the steepest in the BMI group ≤25 kg/m^2^. Looking at the FI level demarcating the “frail” state, the offspring of mothers in the middle BMI category (BMI 25–30 kg/m^2^) reached this level on average 5 years later (75 vs 70 years) than the offspring of mothers in the group with the highest BMI (BMI > 30 kg/m^2^).

### Childhood SES, Wartime Separation From Both Parents in Childhood, and the Level and Rate of Change in FI Levels From Midlife Into Old Age

Children whose fathers were “manual workers” were estimated to have 2.312% higher (95% CI = 0.889, 3.736; *p* = .002) FI levels in midlife than children of “upper middle class” fathers (Table 2). However, childhood SES was unrelated to the rate of change in FI levels from midlife into old age. Girls who had been separated from their parents during World War II had 3.429% lower FI levels (95% CI = −6.670, −0.189; *p* = .039) in midlife but experienced a 0.211 PP steeper increase (95% CI = 0.009, 0.414; *p* = .041) in FI levels from midlife into old age.

### Body Size and Growth From Infancy to Childhood and the Level and Rate of Change in FI Levels From Midlife Into Old Age

Supplementary Table 5 shows that bigger size at the age of 11 years was associated with a higher FI level in midlife. Per 1 kg greater weight and 1 kg/m^2^ higher BMI at the age of 11 years the FI levels in midlife were 0.104% (95% CI = 0.005, 0.203; *p* = .039) and 0.340% higher (95% CI = 0.041, 0.639; *p* = .026), respectively. More rapid weight and BMI gain between the ages of 2 and 7 years were associated with a higher FI level in midlife, the estimates being 0.637% higher (95% CI = 0.099, 1.175; *p* = .021) and 0.839% higher (95% CI = 0.308, 1.369; *p* = .002) for weight and BMI gain, respectively. We observed no associations between other individual measurements of size or growth and the FI level or its rate of change from midlife into old age.

## Discussion

We studied the association between early-life factors and frailty measured over 3 measurement occasions and found evidence of developmental factors contributing to differences in frailty from midlife into old age (ages 57–84 years). In this study, the offspring of mothers with a higher BMI became frail earlier than the offspring of mothers with a lower BMI. Those weighing less at birth or those with a shorter duration of gestation became frail earlier than those weighing more or with a longer duration of gestation. Moreover, we found evidence of bigger childhood size, accelerated growth, and low SES associating with a higher FI level at age 57 years. Together, the findings constitute evidence of disparities in age-related deficit accumulation from midlife into old age which can be tracked back to developmental factors.

In the attempt to derive scales that identify situations where an individual’s “biological age” exceeds their chronological age, of 9 scales considered the FI showed the largest mortality risk prediction alongside methylation age measures (29). In this way, a faster rate of change observed in an FI may also relate to a more rapid occurrence of age-related changes. The present study provides evidence that heterogeneity in the rates of aging may be traceable to factors originating in utero, a question posed by researchers more than 20 years ago (30).

Developmental programming, in which perturbations in prenatal life may irrevocably alter the developing fetus, has been shown to affect later health broadly (9). We have previously reported that a small size at birth conferred increased risk of phenotypic frailty in the seventh decade of life in this cohort (6), as did wartime separation from both parents and certain childhood growth characteristics among boys (4,5). Now extended to the rate of change in an FI from midlife into old age, the findings stress the potential relevance of early-life factors on deficit accumulation. Composed of a range of conditions, symptoms, aspects of functioning, laboratory, and clinical measurements, the FI aims at capturing a diverse measure of frailty (11). Using lower birth weight as an indicator of less beneficial conditions in utero, studies have shown associations between lower birth weight and increased risk of cardiovascular disease (31), diabetes (32), depression (33), poorer physical functioning (34), less optimal body composition (35), lower grip strength (36), and increased mortality from all causes (37), among others. Through other than accumulating deficits themselves, it is also possible that recovery from acquired deficits, for example, the probability of improving functional ability or recover from abnormal laboratory values, may be hindered among these individuals, through physical, genetic, and epigenetic mechanisms (9).

In the present study, the participants’ mothers’ body size was assessed on admission to labor. Obese mothers have higher levels of inflammatory and metabolic parameters (38) and animal studies show epigenetic alterations in offspring of mothers following a high-fat diet (39). A higher maternal BMI has been associated with offspring risk of developing cancer, coronary heart disease, diabetes, and stroke (40). Reynolds et al. observed the odds of offspring death to follow a U-shaped curve, in which the risk of death was higher among mothers with low or high prepregnancy BMI (41). Associations between maternal BMI and performance measures in old age have described sex differences (21–23). Only the male offspring of undernourished mothers had poorer grip strength and physical performance at the age of 68 years (21). A higher maternal BMI has been associated with poorer offspring physical and mental functioning in the sixth decade of life in men (22). The U-shaped association between maternal BMI and offspring total physical activity in the sixth decade was observed only among women (23). However, we observed no sex difference regarding the rate of change in frailty in the present study. Moreover, the observed association persisted after adjustment for body size at birth, suggesting at least partially independent effect of maternal BMI on offspring risk of frailty.

The study participants have been followed through childhood, with information on growth and socioeconomic circumstances. Corroborating a previous finding (8), lower SES in childhood was associated with a higher FI level in midlife. However, SES in childhood was unrelated to the rate of change in FI levels from midlife into old age. Previous findings of accelerated growth in childhood (4) and wartime separation from both parents (5) as risk factors of phenotypic frailty in men are partially confirmed in the present study. While we found evidence of a sex interaction between wartime separation and FI level, in that girls had lower FI levels in midlife, we found no evidence of a sex interaction regarding early size or growth. More rapid weight and BMI growth in early childhood (2–7 years) and greater body size at age 11 years were associated with a greater FI level in both sexes combined. The negative health consequences of accelerated early growth are posited to result from disproportionately high fat mass relative to muscle mass, potentially affecting tissue metabolism, for example, through insulin resistance (13).

### Strengths and Weaknesses

We used unique early-life data from gestation to childhood and studied their associations with an FI measured on 3 occasions from midlife into old age. The FI has shown discriminative ability in predicting adverse outcomes over the frailty phenotype definition (17,42), most likely because the FI is a continuous multidimensional measure, whereas the phenotype captures physical frailty, which when used previously in this cohort yielded a low prevalence of frailty of 3.6% (6). While the HBCS–FI exceeded the minimum of 30 deficits, we omitted deficits on cognitive performance, other laboratory parameters, and sensory problems due to insufficient data. The long interval between the exposure and outcome may have resulted in residual confounding unaccounted for in the present analysis. Approximately every sixth participant had died by the last FI measurement occasion, a group likely represented by higher levels of frailty, and potentially undermine associations found in the present study. However, included and excluded participants shared largely similar early-life characteristics. Assuming not missing at random sample attrition did not significantly alter our results, giving little support that sample attrition threatened the validity of our results. Mother’s BMI was assessed on admission to labor and no information on prepregnancy weight or gestational weight gain was available. The data come from individuals born in Helsinki, Finland, between 1934 and 1944. Thus, the results should be interpreted with caution.

## Conclusion

In conclusion, developmental factors, including maternal BMI and birth weight, associated with age-related deficit accumulation from midlife into old age. Consequently, the course and trajectory of frailty may in part be influenced by early developmental factors. Individuals who had perturbances in their early life may represent a group requiring special focus to prevent the onset and progression of frailty. Efforts to improve the health and well-being of women of childbearing age and their newborn babies may help narrow down disparities in age-related deficit accumulation across older age.

## Supplementary Material

glac007_suppl_Supplementary_Material
