# Supplementary material for: Early-Life Factors as Predictors of Age-Associated Deficit Accumulation Across 17 Years From Midlife Into Old Age
Source: J Gerontol A Biol Sci Med Sci. 2022 Jan 9;77(11):2281–7. doi: 10.1093/gerona/glac007 (PMC9678199; doi:10.1093/gerona/glac007)
Supplement: glac007_suppl_Supplementary_Material [file glac007_suppl_supplementary_material.pdf]

Supplementary material legend

Supplementary Table 1. List of the 41 variables included in the frailty index in the Helsinki Birth Cohort Study.

Supplementary Table 2. Measurements of size from infancy to childhood of participants participating in the baseline clinical visit.

Supplementary Table 3. Characteristics of participants participating in baseline clinical measurements according to status at the follow-up visit.

Supplementary Table 4. One-unit increases in early life factors predicting point estimates of the FI level at age 57 years and the rate of change in FI levels from midlife into old age assuming missing not at random (MNAR).

Supplementary Table 5. Measurements of size and growth from infancy to childhood predicting point estimates of the FI level at age 57 years and the rate of change in FI levels from midlife into old age.

Supplementary Table 1. List of the 41 variables included in the frailty index in the Helsinki Birth Cohort Study.

	Variable name	Scoring
1	Self-reported angina pectoris as diagnosed by a doctor[a]	Yes=1; no=0
2	Self-reported asthma as diagnosed by a doctor[a], special reimbursement for obstructive airway disease medication[b]	Yes=1; no=0
3	Self-reported cancer as diagnosed by a doctor[a]	Yes=1; no=0
4	Self-reported claudication as diagnosed by a doctor[a]	Yes=1; no=0
5	Special reimbursement for antiarrhythmic medication[b]	Yes=1; no=0
6	Self-reported depression by a doctor[a]	Yes=1; no=0
7	Self-reported diabetes as diagnosed by a doctor[a], special reimbursement for diabetes medication[b]	Yes=1; no=0
8	Self-reported emphysema as diagnosed by a doctor[a]	Yes=1; no=0
9	Special reimbursement for glaucoma medication[b]	Yes=1; no=0
10	Self-reported heart failure as diagnosed by a doctor[a]	Yes=1; no=0
11	Self-reported hypertension as diagnosed by a doctor[a], special reimbursement for antihypertensive drugs[b]	Yes=1; no=0
12	Self-reported myocardial infarct as diagnosed by a doctor[a]	Yes=1; no=0
13	Self-reported osteoporosis as diagnosed by a doctor[a]	Yes=1; no=0
14	Self-reported stroke as diagnosed by a doctor[a]	Yes=1; no=0
15	General health[c]	Poor=1; fair=0.75; good=0.50; very good=0.25; excellent=0

16	Health compared to one year ago[c]	Worse=1; same/better=0
17	Health limits vigorous activities[c]	Yes=1; yes a bit=0.50; no=0
18	Health limits moderate activities[c]	Yes=1; yes a bit=0.50; no=0
19	Health limits lifting or carrying groceries[c]	Yes=1; yes a bit=0.50; no=0
20	Health limits climbing several flights of stairs[c]	Yes=1; yes a bit=0.50; no=0
21	Health limits bending, kneeling, or stooping[c]	Yes=1; yes a bit=0.50; no=0
22	Health limits walking more than a kilometre[c]	Yes=1; yes a bit=0.50; no=0
23	Health limits walking more than 100 metres[c]	Yes=1; yes a bit=0.50; no=0
24	Health limits bathing or dressing[c]	Yes=1; yes a bit=0.50; no=0
25	Physical health limits the kind of work or other activities[c]	Yes=1; no=0
26	Bodily pain[c]	Severe/very severe=1; moderate=0.75; mild=0.50; very mild=0.25; no pain=0
27	Feel full of life[c]	None=1; some/little=0.50; all/most/good bit=0
28	Feel tired[c]	All/most/good bit=1; some/little=0.50; none=0
29	Body mass index (kg/m^2)	<18.5 or ≥ 30=1; >25 and <30=0.50; ≥18.5 and ≤25=0
30	Waist circumference to hip circumference ratio	≥0.95 for men or ≥0.90 for women=1; ≥0.90 and <0.95 for men or ≥0.85 and <0.90 for women=0.50; <0.90 for men or <0.85 for women=0
31	Heart rate (bpm)	<60 or >100=1; ≥60 and ≤100=0

32	Systolic blood pressure measured to be ≥ 160 mmHg or diastolic blood pressure measured to be ≥ 100 mmHg	Yes=1; no=0
33	Abnormal fasting glucose (≥ 6.1 mmol/l)[d]	Yes=1; no=0
34	High total cholesterol level (> 5.0 mmol/l)[d]	Yes=1; no=0
35	Low high-density lipoprotein level (men < 1.00 mmol/l, women < 1.20 mmol/l)[d]	Yes=1; no=0
36	Abnormal alanine amino transferase / aspartate transaminase level (ALT > 50 U/l for men and > 35 U/l for women; AST > 45 U/l for men and > 35 U/l for women)[d]	Yes=1; no=0
37	Weekly metabolic equivalent of physical activity in hours (METh/week)	Lowest quintile stratified by sex=1; other=0
38	Less interested in other people than before[e]	Yes=1; no=0
39	Changes in sleeping pattern[e]	Yes=1; more tired in the morning=0.50; sleep as well as before=0
40	Changes in appetite[e]	Less than usual=1; no change=0
41	Weight loss[e]	2.5 kg or greater=1; weight has been stable=0

Note. [a]Assessed using questionnaires at clinical baseline and follow-up visits; [b]prescription medicines reimbursed out of National Health Insurance until the year 2017; [c]from the RAND-36/SF-36 questionnaire (1); [d]cut-offs indicating abnormal test results; [e]from the Beck Depression Inventory (BDI) (2) questionnaire.

1. RAND. The RAND 36-Item Health Survey The RAND 36-Item Health Survey. *Heal*

(San Fr. 1992;2(March):91-97. doi:10.1002/hec.4730020305.

2. Beck AT, Steer RA BG. *Manual for the Beck Depression Inventory-II*. San Antonio TX: Psychological Corporation; 1996.

Supplementary Table 2. Measurements of size from infancy to childhood of participants participating in the baseline clinical visit.

	Total population		Women		Men	
	N	Mean (SD)	N	Mean (SD)	N	Mean (SD)
**Measurements of size from infancy to childhood**						
Height (cm) at age						
1 year	2001	75.7 (2.6)	1074	74.9 (2.5)	927	76.6 (2.5)
2 years	2001	86.1 (3.1)	1074	85.5 (3.0)	927	86.8 (3.0)
7 years	1918	120.5 (4.7)	1025	120.0 (4.7)	893	121.0 (4.8)
11 years	1922	141.7 (6.3)	1031	141.6 (6.6)	891	141.7 (5.9)
Weight (kg) at age						
1 year	2003	10.2 (1.1)	1075	9.9 (1.0)	928	10.4 (1.0)
2 years	2003	12.1 (1.2)	1075	11.9 (1.1)	928	12.4 (1.1)
7 years	1918	22.4 (2.8)	1025	22.2 (2.9)	893	22.6 (2.6)
11 years	1923	34.1 (5.2)	1032	34.4 (5.7)	891	33.9 (4.6)
Body mass index (kg/m^2) at age						
1 year	2001	17.7 (1.4)	1074	17.6 (1.4)	927	17.8 (1.3)
2 years	2001	16.5 (1.2)	1074	16.4 (1.2)	927	16.6 (1.2)
7 years	1916	15.5 (1.2)	1024	15.5 (1.3)	892	15.5 (1.1)
11 years	1919	17.0 (1.7)	1029	17.1 (1.9)	890	16.8 (1.5)

Note. SD=standard deviation.

Supplementary Table 3. Characteristics of participants participating in baseline clinical measurements according to status at the follow-up visit.

	**Invited to clinical follow-up visit** n=1404 Mean (SD)	**Declined/no contact/lived further away** n=448 Mean (SD)	**Died** n=151 Mean (SD)	**P**
**Birth factors**				
Weight (kg)[b]	3.41 (0.48)	3.38 (0.50)	3.48 (0.54)	0.032
Length (cm)	50.3 (1.9)	50.2 (2.0)	50.5 (2.2)	0.160
BMI (kg/m^2)	13.41 (1.23)	13.37 (1.26)	13.56 (1.29)	0.059
Gestational age (weeks)	40.0 (1.6)	40.1 (1.5)	40.1 (1.5)	0.285
Maternal BMI (kg/m^2)	26.6 (2.9)	26.4 (2.8)	26.8 (3.1)	0.136
**Wartime separation from both parents during World War II**				
Separated, N (%)[c]	164 (13.0)	80 (19.3)	25 (18.1)	0.004
**Childhood SES**				0.554
Manual worker, N (%)	828 (59.3)	275 (62.1)	90 (60.4)	
Lower middle class, N (%)	315 (22.5)	102 (23.0)	36 (24.2)	
Upper middle class, N (%)	254 (18.2)	66 (14.9)	23 (15.4)	
**Adult SES**				0.003

Manual worker, N (%)	441 (31.4)	172 (38.5)	58 (38.4)	
Self-employed, N (%)	125 (8.9)	42 (9.4)	20 (13.2)	
Lower official, N (%)	633 (45.1)	164 (36.7)	61 (40.4)	
Upper official, N (%)	205 (14.6)	69 (15.4)	12 (7.9)	
**Participant characteristics assessed in early old age**				
Age (years)	61.4 (2.9)	61.7 (3.0)	62.1 (3.1)	0.023
**Frailty index**				
Baseline measurement occasion in 2001-2004[a]	0.19 (0.09)	0.22 (0.11)	0.25 (0.12)	<0.001

Note. SD=standard deviation; BMI=body mass index; SES=socioeconomic status.

[a]n=1995. [b]Pairwise group comparisons showed that dead participants were born bigger than participants who were declined/were not contacted/lived far away (p=0.026). [c]Pairwise group comparisons showed that fewer invited participants had been separated than dead and participants who were declined/were not contacted/lived far away (p-values<0.05).

Supplementary Table 4. One-unit increases in early life factors predicting point estimates of the FI level at age 57 years and the rate of change in FI levels from midlife into old age assuming missing not at random (MNAR).

	**Level** [a]	**95 % CI**	**P**	**Rate of change** [b]	**95 % CI**	**P**
**Early life factor**						
**Birth factors**						
Weight (kg)[c]	0.008	-0.011, 0.127	0.898	-0.008	-0.015, -0.001	0.043
Length (cm)[c]	0.028	-0.262, 0.317	0.852	-0.014	-0.033, 0.004	0.127
BMI (kg/m^2)[c]	-0.049	-0.482, 0.383	0.823	-0.027	-0.055, 0.001	0.061
Gestational age (weeks)	0.136	-0.189, 0.461	0.412	-0.017	-0.038, 0.003	0.097
Maternal BMI (kg/m^2)[c]	0.268	0.073, 0.463	0.007	-0.010	-0.023, 0.002	0.102
**Wartime separation from both parents during World War II**[e]						
Separated	2.692	0.268, 5.115	0.029	-0.053	-0.207, 0.101	0.503
Separated × female sex	-4.004	-7.209, -0.799	0.014	0.207	0.003, 0.410	0.047
**Childhood socioeconomic status**[e]						
Manual worker	1.815	0.393, 3.238	0.012	-0.072	-0.156, 0.012	0.093
Lower middle class	1.282	-0.304, 2.868	0.113	0.018	-0.115, 0.078	0.709
Upper middle class	Ref.			Ref.		

Note. CI=confidence interval; BMI=body mass index.

[a]In FI × 100 units, which correspond percentage increases/decreases in FI levels at age 57 years (mean FI level at age 57 years was 0.186).

[b]In percentage points per year from midlife into old age (mean annual rate of change in FI levels from midlife into old age was 0.34 percent/year).

[c]Model adjusted with age, sex, gestational age, childhood and adult socioeconomic status (SES).

[e]Model adjusted with age, sex, and adult SES

Supplementary Table 5. Measurements of size and growth from infancy to childhood predicting point estimates of the FI level at age 57 years and the rate of change in FI levels from midlife into old age.

	Level [a]	95 % CI	P	Rate of change [b]	95 % CI	P
**Measurements of size from infancy to childhood[c]**						
Height (cm) at age						
2 years	0.068	-0.102, 0.238	0.434	-0.008	-0.018, 0.003	0.140
7 years	0.009	-0.103, 0.121	0.875	0.001	-0.007, 0.007	0.930
11 years	0.010	-0.073, 0.094	0.807	-0.001	-0.006, 0.004	0.721
Weight (kg) at age						
2 years	-0.197	-0.650, 0.257	0.396	-0.017	-0.045, 0.011	0.241
7 years	0.106	-0.085, 0.296	0.277	-0.004	-0.017, 0.008	0.474
11 years	0.104	0.005, 0.203	0.039	-0.003	-0.010, 0.003	0.284
Body mass index (kg/m^2) at age						
2 years	-0.379	-0.796, 0.038	0.075	0.002	-0.024, 0.027	0.909
7 years	0.333	-0.084, 0.749	0.118	-0.018	-0.045, 0.008	0.182
11 years	0.340	0.041, 0.639	0.026	-0.011	-0.030, 0.008	0.250

	Level [a]	95 % CI	P	Rate of change [b]	95 % CI	P
**Growth from infancy to childhood[c]**						
From birth to 2 years						

Weight growth	-0.178	-0.713, 0.356	0.514	-0.001	-0.033, 0.033	0.992
Height growth	0.403	-0.137, 0.942	0.145	-0.014	-0.047, 0.019	0.417
BMI growth	-0.463	-0.990, 0.064	0.086	0.011	-0.021, 0.044	0.506
From 2 to 7 years						
Weight growth	0.637	0.099, 1.175	0.021	0.003	-0.031, 0.037	0.860
Height growth	-0.231	-0.774, 0.313	0.407	0.027	-0.008, 0.062	0.129
BMI growth	0.839	0.308, 1.369	0.002	-0.022	-0.056, 0.012	0.197
From 7 to 11 years						
Weight growth	0.503	-0.028, 1.034	0.063	-0.017	-0.050, 0.016	0.310
Height growth	0.097	-0.444, 0.638	0.727	-0.017	-0.050, 0.016	0.309
BMI growth	0.467	-0.067, 1.001	0.087	-0.007	-0.040, 0.027	0.689

Note. CI=confidence interval; BMI=body mass index.

[a]In FI × 100 units, which correspond to percent increases/decreases in FI level at age 57 years (mean FI level at age 57 years was 0.186).

[b]In percentage points per year from midlife into old age (mean annual rate of change in FI levels from midlife into old age was 0.34 percent/year).

[c]Model adjusted with sex, childhood and adult socioeconomic status (SES).
